# Social Engagement, Health, and Changes in Occupational Status: Analysis of the Korean Longitudinal Study of Ageing (KLoSA)

**DOI:** 10.1371/journal.pone.0046500

**Published:** 2012-10-02

**Authors:** Jin-young Min, Kyung-jong Lee, Jae-beom Park, Sung-il Cho, Shin-goo Park, Kyoungbok Min

**Affiliations:** 1 Institute of Health and Environment, Seoul National University, Chongno-gu Yongeun-dong 28, Seoul, Republic of Korea; 2 Department of Occupational and Environmental Medicine, Ajou University School of Medicine, Suwon, Republic of Korea; 3 Department of Epidemiology, School of Public Health and Institute of Health and Environment, Seoul National University, Seoul, Republic of Korea; 4 Department of Occupational and Environmental Medicine, Inha University Hospital, Incheon, Republic of Korea; Wayne State University, United States of America

## Abstract

**Background:**

We focused on whether changes in the occupational status of older male adults can be influenced by social engagement and health status measured at the baseline.

**Methods:**

This study used a sample of the Korean Longitudinal Study of Aging (KLoSA), and the study population was restricted to 1.531 men who were aged 55 to 80 years at the 2006 baseline survey and participated in the second survey in 2008. Social engagement and health status, measured by the number of chronic diseases, grip strength, and depressive symptoms as well as covariates (age, marital status, educational level, and household income) were based on data from the 2006 baseline survey. Occupational engagement over the first and second survey was divided into four categories: ‘consistently employed’ (n = 892), ‘employed-unemployed’ (n = 152), ‘unemployed-employed’ (n = 138), and ‘consistently unemployed’ (n = 349).

**Results:**

In the multinomial model, the ‘consistently employed’ and ‘unemployed-employed’ groups had significantly higher social engagement (1.19 and 1.32 times, respectively) than the referent. The number of chronic diseases was significantly associated with four occupational changes, and the ‘unemployed-employed’ had the fewest chronic conditions.

**Conclusion:**

Our finding suggests that social engagement and health status are likely to affect opportunities to continue working or to start working for older male adults.

## Introduction

Occupation is central to fulfillment and socialization in people’s lives. From personal reasons to social and economic outcomes, individuals place values on their occupation, and this value has a direct and indirect effect on many aspects of human of well-being.

In particular, the significance of occupation might be more distinct among older people, because their vulnerabilities in health, employment, and social ties emphasize the positive aspects of work (i.e., economic independence, social support, and emotional recognition) [1]. Thus, occupation can be considered as a key component in successful aging [2]. Furthermore, a decline in the fertility rate and an increases in longevity are contributing to the population becoming older, and social expenditures for medical, welfare, and security for the elderly are increasing. Thus, the importance of occupation is extending beyond private needs to a broader social context [3].

Many studies have highlighted the role of occupation in determining health and economic well-being among older people. Key findings have shown that occupational experience is associated with health status and cognitive functions [4]–[8]. There is increasing evidence to suggest the relevance of retirement or involuntary job loss on subsequent health and economic outcomes among older adults [9]–[11]. Strategies to employ older persons continue to be established [3].

Despite multilateral efforts, unresolved issues remain in the relationship between aging and occupation. For example, although a recent trend in the labor market has encouraged the employment of older workers, individual’s capacity for employment may vary. The differences are likely to be derived from individual’s own backgrounds and abilities. Therefore, what are the characteristics of older people that predict their success or failure in securing employment? In this study, we focused on social engagement and health status as major determinants and investigated whether these factors influence occupational status in older male adults. Several studies have shown beneficial effects social engagement on physical and mental health outcomes and on slowing cognitive decline among older adults [12]–[14]. Considering that improving physical and mental fitness may be essential for older adults to perform their job functions, social engagement may be a significant indicator to continue working or to start working for older adults. Occupational status was evaluated by changes in occupational engagement over the study periods. The four employment groups used were ‘consistently employed’, ‘employed and unemployed’, ‘unemployed and employed’ and ‘consistently unemployed’.

## Methods

### Data Collection and Participants

This study uses a sample from the first and second wave data of the Korean Longitudinal Study of Aging (KLoSA) [15]. The KLoSA is being conducted by the Korea Labor Institute to collect the basic data needed to devise and implement effective social and economic policies that address emerging trends related to population aging. It will be repeated every even-numbered year.

This original KLoSA study population comprised South Korean adults aged 45 years or older who lived in 15 large administrative areas. In the first baseline survey in 2006, 10,254 individuals in 6,171 households (1.7 per household) were interviewed using the Computer-Assisted Personal Interviewing method. The second survey in 2008 followed up with 8,688 subjects who represented 86.9% of the original panel, with the exception of 254 decedents; this group was composed of 3767 men and 4921 women.

For this study, we restricted our population to men because the majority of women in this cohort did not work. The KLoSA sample initially included 2,535 men who were aged 55 to 80 years old at the 2006 baseline survey and participated in the 2008 second survey. Of these participants, we additionally excluded 177 subjects with no occupational information and 826 subjects who wanted to retire or were retired voluntarily, leaving a final sample of 1531older male adults. We excluded women Majority of women in this cohort did not work.

Korean Longitudinal Study of Aging (KLoSA) is a National public database (website: http://www.kli.re.kr/klosa/en/about/introduce.jsp). The data had ID but was anonymized. The system of identities was totally designated to protect subjects. Study protocol was approved by ethics review board of the Ajou University Hospital. The review was conducted by the rightful process. Participants had to read and sign an agreement form before participating in the KLOSA study, and agreed to be used in further scientific research.

### Changes in Occupational Engagement Over the Two-year Period

We divided changes in occupational engagement between the first and second survey into four categories: ‘consistently employed’, ‘employed-unemployed’, ‘unemployed-employed’, and ‘consistently unemployed’. ‘Consistently employed’ (n = 892) included people who had been consistently working during the two-year period, and ‘Consistently unemployed’ (n = 349) included people who had never worked during these years. ‘Employed-unemployed’ (n = 152) included people who had worked the first year, but had not worked since, and ‘unemployed-employed’ (n = 138) included people who did not work during first year, but started working at the some point during the two-year period.

### Measurements

All variables related to subjects’ characteristics, except occupation, were based on data from the 2006 baseline survey.

#### Social engagement

Social engagement’ included formal social engagements and social interactions with other friends and relatives. The presence of formal social engagement was measured with the following question: “I am participating in ‘religious organizations’; ‘fraternal organizations’; ‘leisure, culture, or sports clubs’; ‘school or family reunions’; ‘voluntary or charity work’; ‘political organizations’, or ‘others’. Each item was coded ‘Yes’ or ‘No’. In addition, social interaction was measured based on the question, “Do you have close friends, relatives, or neighborhood friends? How often do you get together with them?”. If subjects responded with either of the two items, ‘I hardly ever see them in a year’ or ‘There is no such person’, they were assumed as having no social interaction. Other responses (i.e., almost every day, once a week, two or three times a week, once a month, every two weeks, once or twice a year, three or five times a year, and once every two months) lead to the assumption that they have social interaction. Positive responses on questions pertaining to formal social engagements or social interaction were summed.

#### Health status

Health status was measured by grip strength, depression scale, and the number of chronic diseases. Grip strength was measured by a handgrip dynamometer (Model number: NO6103, Manufacturer: TANITA, Japan). The test was performed in a sitting position with the elbow flexed at 90° on both the right and left sides. The mean strength was calculated from grip strength on both sides. If subjects could not perform the grip test, the value from the other hand was used for the analysis. We evaluated depression using the 10-item short form of the Center for Epidemiological Studies Depression tool (CES-D10). The CES-D10 is a screening tool for assessing depressive symptoms, and it contains two positive items (feel pretty good, overall satisfied), and eight negative items (loss of interest, trouble concentrating, feeling depressed, feeling tried or having low energy, being afraid of something, having trouble falling asleep, feeling alone, and feeling down on yourself) [16]. The score is assigned a value of zero, one, two, or three, (reversed for positive items), and higher scores indicates greater depressive symptoms. The number of chronic diseases was measured by self-reported disease histories. Respondents reported one or more physician-diagnosed diseases, including hypertension, diabetes mellitus, cardiac disorders, gastrointestinal diseases, arthritis, cancer, lung disease, and stroke.

#### Covariates

We used age, marital status (married, widow/divorced, unmarried), educational level (elementary school or less, middle school, high school, college or more), and household income as covariates.

### Statistical Analysis

**Figure 1:**
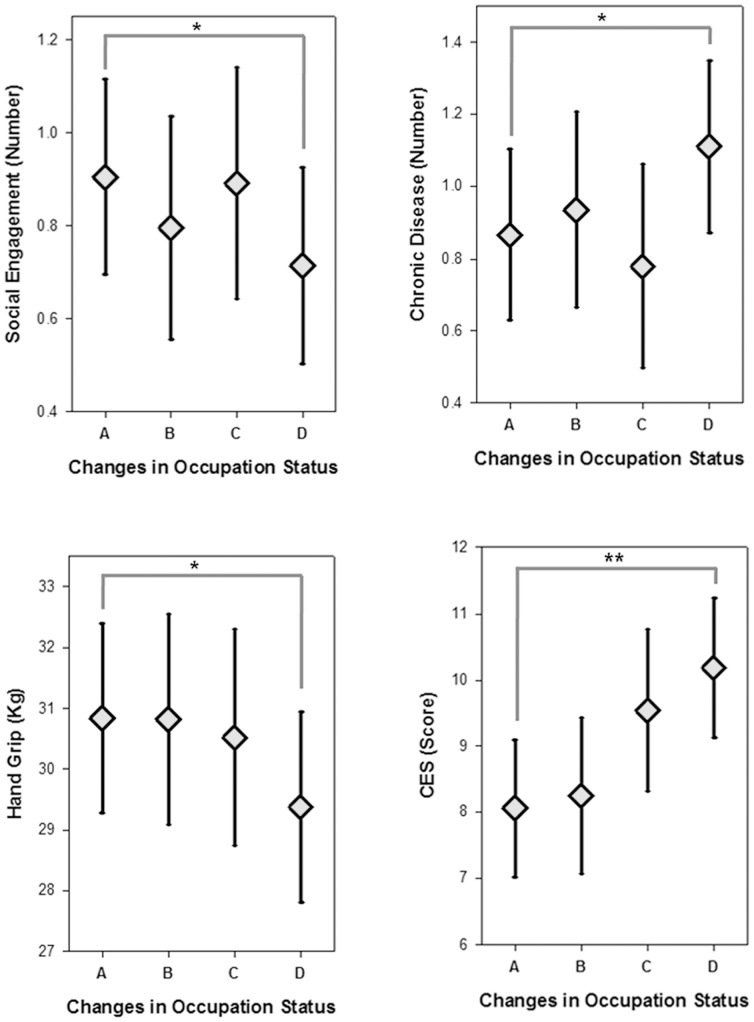
Least squares means and standard errors for social engagement, the number of chronic diseases, grip strength, and CES-D according to changes in occupational status; A-‘Consistently employed’, B-‘Employed-unemployed’, C-‘Unemployed-employed’, D-‘Consistently unemployed’.

Employment group was divided into four categories: ‘consistently employed’, ‘employed-unemployed’, ‘unemployed-employed’, and ‘consistently unemployed’. We listed frequencies and means (±SD) of the baseline characteristics of the participants and compared them to the four categories using chi-squared tests and ANOVA (Table 1). To explore whether changes in occupational engagement were associated with social engagement, grip strength, CES-D, and the presence of chronic diseases, least square (LS) means and standard errors were calculated according to the four occupational categories, controlling for age, marital status, educational level, and household income (Figure 1). Subsequently, we performed a multinomial logit regression model to estimate the relationship between social engagement, health status, and changes in occupational engagements over the two years. The logit model was adjusted for age, marital status, educational level, and household income: age and income were entered into the model as continuous variables, and all other covariates were entered as categorical variables. The odds ratio (OR) was obtained by taking the exponent of the estimated coefficients. Statistical analyses were carried out with SAS software, version 9.1 (SAS institute, Inc., Cary, North Carolina).

**Table 1:** General characteristics at the baseline survey of study population according to changes in occupational status.

		‘Consistentlyemployed’ (n = 892)	‘Employed andUnemployed’ (n = 152)	‘Unemployed andemployed’ (n = 138)	‘Consistentlyunemployed’ (n = 349)	*p*-value
Age (years)		62.37 (5.66)	64.04 (5.41)	63.99 (6.17)	67.64 (6.36)	<0.0001
Social engagement		2.15 (1.06)	2.01 (1.08)	1.98 (0.99)	1.66 (1.02)	<0.0001
Chronic disease		0.76 (0.93)	0.88 (1.01)	0.72 (1.09)	1.14 (1.11)	<0.0001
Hand grip strength		32.73 (6.05)	32.24 (5.63)	30.92 (6.49)	28.46 (6.49)	<0.0001
CES-D		5.10 (3.80)	5.35 (4.30)	7.18 (5.52)	8.04 (4.92)	<0.0001
Income (won)		2128.28 (2104.74)	2278.22 (2790.33)	1032.81 (1552.74)	1160.41 (1450.75)	<0.0001
Marital status, n (%)						
Married		845 (94.73)	143 (94.08)	124 (89.86)	308 (88.25)	0.0001
Widowed or divorced		46 (5.16)	7 (4.61)	14 (10.14)	35 (10.03)	
Never married		1 (0.11)	2 (1.32)	0 (0)	6 (1.72)	
Education, n (%)						
Elementary school or less		303 (33.97)	52 (34.21)	76 (55.07)	194 (55.58)	<0.0001
Middle school		206 (23.09)	32 (21.05)	18 (13.04)	68 (19.48)	
High school		265 (29.71)	49 (32.24)	38 (27.54)	58 (16.62)	
College or more		118 (13.23)	19 (12.5)	6 (4.35)	29 (8.31)	

Data represent mean values (SD).

## Results

Table 1 showed the general characteristics upon the baseline survey of the study population according to occupational changes. About half of the subjects were included in the ‘consistently employed’ group, and only 19 percent experienced occupational changes. The mean age of across all groups was 65.7 years old, and the ‘consistently unemployed’ group was the oldest. The average number of social engagement was across all groups 2.0, and the ‘consistently unemployed’ reported the lowest social engagement. The ‘consistently unemployed’ group also reported the largest number of chronic diseases (1.14), whereas the ‘consistently employed’ had the fewest chronic diseases (0.76). Subjects who worked at the baseline survey had higher grip strength and fewer depressive symptoms than those who did not work at the baseline. The mean income of the ‘consistently employed’ and ‘employed and unemployed’ was higher than the ‘consistently unemployed’ and ‘unemployed and employed’. The proportions of married men (88–95%), widowed/divorced, and never married, in that order, among four groups were the highest. About half of the ‘unemployed and employed’ and ‘consistently unemployed’ and were in the least-educated categories, whereas nearly one-third of participants in the other groups had the lowest level of education. The distributions of all variables differed significantly among the four groups.

Figure 1 showed the least square means of social engagement, the number of chronic diseases, grip strength, and depressive symptoms, according to occupational changes. Each mean value was adjusted by age, income, marital status, and educational level. Social engagement varied by employment group: it was highest in the ‘unemployed and employed’ group, followed by ‘consistently employed’, ‘employed and unemployed’ and ‘consistently unemployed’, in that order. The number of chronic diseases was lowest in the ‘unemployed and employed’ group and highest in the ‘consistently unemployed’ group. Subjects with a job at the baseline survey had relatively stronger hand-grip strength and less depressive symptoms than those without a job at the time. The ‘consistently unemployed’ were the worst in terms of social engagement and health status compared with the other employment groups.

To confirm the role that social engagement and health play in occupational changes, we did multinomial logit analysis controlling for variables, and the results are shown in Table 2. In the multinomial logit model, a value less than one explains that social engagement and health status reduce the likelihood of the specific changes in occupational status, compared with the ‘consistently unemployed’ that was used as a reference. All variables, with the exception of changes in occupational status, were measured at the baseline survey, and occupational changes included the changes for two study periods. According to this model, the ‘consistently employed’ and ‘unemployed and employed’ groups had significantly more instance of social engagement (1.32 and 1.39 times, respectively) than the reference group. After adjusting for health outcomes including the number of chronic diseases, the hand grip strength, and depressive symptoms, the results remained robust: the corresponding ORs (95% CIs) were 1.19 (95% CI, 1.01–1.39) for ‘consistently employed’ and 1.32 (95% CI, 1.04–1.68) for ‘unemployed-employed’. The number of chronic diseases was significantly associated with all four changes in occupational status, and the ‘unemployed and employed’ group had the fewest chronic conditions. Subjects who were consistently unemployed had lower levels of hand-grip strength and higher levels of depressive symptoms at baseline compared with the ‘consistently employed’ and ‘employed and unemployed’ groups.

**Table 2:** Estimated impact of social engagement, chronic disease, grip strength, and depressive symptoms on transitions of occupational status.

	OR (95% CI)	*p*-value
Social Engagement		
Social engagement adjusted for age, sex, marriage, and income		
‘Consistently employed’	1.32 (1.14–1.53)	0.0003
‘Employed and Unemployed’	1.19 (0.97–1.47)	0.1021
‘Unemployed and employed’	1.39 (1.12–1.74)	0.0003
‘Consistently unemployed’	1.00 (reference)	
Social engagement further adjusted for health outcomes*		
‘Consistently employed’	1.19 (1.01–1.39)	0.0033
‘Employed and Unemployed’	1.07 (0.86–1.34)	0.5310
‘Unemployed and employed’	1.32 (1.04–1.68)	0.0210
‘Consistently unemployed’	1.00 (reference)	
Chronic Disease**		
‘Consistently employed’	0.83 (0.72–0.94)	0.0049
‘Employed and Unemployed’	0.87 (0.71–1.06)	0.1704
‘Unemployed and employed’	0.74 (0.60–0.92)	0.0076
‘Consistently unemployed’	1.00 (reference)	
Hand Grip strength**		
‘Consistently employed’	1.05 (1.02–1.08)	0.0011
‘Employed and Unemployed’	1.05 (1.01–1.09)	0.0251
‘Unemployed and employed’	1.03 (0.99–1.08)	0.1065
‘Consistently unemployed’	1.00 (reference)	
CES-D**		
‘Consistently employed’	0.90 (0.87–0.93)	<.0001
‘Employed and Unemployed’	0.91 (0.87–0.96)	0.0005
‘Unemployed and employed’	0.97 (0.93–1.01)	0.1051
‘Consistently unemployed’	1.00 (reference)	

* Health outcomes included chronic disease, hand grip strength, and CES-D.

** adjusted by age, sex, marriage, and income.

## Discussion

We explored whether social engagement and health status were associated with changes in occupational status in older male adults. We found that social engagement and chronic conditions affected occupational changes in the future, controlling for potential confounders. Specifically, involvement in social engagement was significantly associated with the possibility to keep working or start working later, and the ‘consistently unemployed’ group showed the lowest level of social engagement. There were small differences in the number of chronic diseases among those who had at least one occupational experience (i.e., ‘consistently employed’, ‘employed and unemployed’, and ‘unemployed and employed’) over the study period, but there was a large difference between the ‘continuously unemployed’ group and the others. Notably, individuals included in the ‘unemployed and employed’ group reported the best social engagement and least chronic conditions, whereas the ‘consistently unemployed’ group had the worst chronic conditions. Thus, in considering social policies that encourage older workers and their personal needs from the perspective of occupational benefits, it is important to consider that older men who are more socially engaged and have fewer few chronic diseases are more likely to enjoy positive changes in occupational status. These factors play a pivotal role in the employment of older adults.

Interpreting the association between grip strength, CES-D, and occupational changes is not straightforward. Grip strength and depressive symptoms may be more affected by whether people have a job during the survey period. For example, employed persons (‘consistently employed’ and ‘employed and unemployed’) had stronger hand grip and lower depressive scores than unemployed persons (‘consistently unemployed’ and ‘unemployed and employed’) at the baseline. However, it is notable that subjects with the weakest grip strength and the most depressive symptoms at the baseline stayed at ‘continuously unemployed’, mirroring our findings with social engagement and chronic diseases in this group. From this result, the impact of grip strength and depressive symptoms on changes in occupational status may be small, but low grip strength and a high level of depressive symptoms may negatively affect one’s ability to secure a job.

Although the exact relationship between social engagement, health status, and occupational change is unclear, we hypothesized that the mechanism may be based on better health, cognition, and emotional support. Social engagement is a major activity of daily life, implying a reciprocal connection between people and their communities. A socially engaging lifestyle has been associated with improved cognitive functioning [17]–[19] and better health [12], [20], [21]. Although findings on the effect of social engagement on cognitive function are contradictory, many researchers have supported the theory that a lack of social interaction engenders more significant cognition decline relative to older people with more social interactions. Berkman et al. (2000) has stressed that social engagement through communication and participation is very useful for stimulating and maintaining cognitive capacities in old age [13]. Social engagement is also related to health issues among older adults; the more social involvement, the lower the risk of mortality [14], [22], disability [12]–[14], [21]–[23], depressive symptoms [24], [25], and loss of motor function [26]. Mendes de Leon et al. (2003) pointed out that involvement in social engagement enables individuals to modify the effects of age-related diseases [23]. Further, greater social engagement can increase emotional support from other people [17], [27]. Thus, benefits from social engagement, such as ‘better health and cognition’, and ‘improved emotional support’, might affect changes in occupational status of the elderly. In our study, social engagement may have conferred advantages to subjects who had not worked during the first year but started to work during the two-year study period.

The impact of health status on changes in occupational engagement can be understood through a similar relationship between social engagement and occupational changes. Several studies have found that a reduction in hand-grip strength can predict increased disability, cognitive decline, health complications, and mortality [28]–[32]. High levels of depressive symptoms and chronic conditions are also considered as independent risk factors for these events [33]–[35]. It is widely recognized that health status can be influenced by social and emotional supports [13], [36].

In light of established circumstances, we cannot conclusively prove how social engagement and health status affected occupational status in the current study. While the positive influences of social engagement, strong grip strength, low level of depressive symptoms, and few chromic diseases are not exclusive, the benefit likely stems from a combination of these variables.

The strengths of this study lie in its prospective design, which allowed us to assess the effect of social engagement and health status at baseline and chart further changes in occupational status. Additionally, we regarded occupational status as an outcome variable rather than a cause; this approach differs from previous studies that have evaluated the direct or indirect effect of occupation on health-related problems and treated occupation as a social determinant of health [4]–[8]. In response to the demands of the demographic shift caused by low fertility rates and increased longevity in adults, policy makers in many countries have set an agenda to increase the participation of older people in the labor market. The social welfare system and the labor market have primary responsibility for effectiveness from a policy perspective, but an older individual’s choice to continue, quit, or start working may have close links with his or her capability and potential. Therefore, it is important to recognize individual-level factors that affect employment of older people so that the policies are appropriate for both older adults and society. Here, we reported social engagement and health status as important individual factors that influence changes in the occupational status of older men, especially in subjects who are going to start work.

Despite the advantages of this study, several limitations should be considered when the results are interpreted. First, the period of two years for changes in occupational status might not be sufficient to reflect overall changes. However, occupational periods of older adults may be relatively short due to individual health problems or changeable working environments. A rather short time of occupational changes may be practical for the type of analysis undertaken in this study. Second, because changes in occupational status were estimated by only one question (i.e., whether he/she is engaged in work) in the two surveys, we cannot capture changes in occupational status that occurred in the middle of the first wave and the second wave. This is likely to reflect only phenomena that occurred at the survey time. For example, one person worked at the first wave and stopped working thereafter, then restarted work just before the second wave. This person would be categorized as ‘consistently employed’, even though he did not work continuously. Other complex changes can possibly occur. However, in our sample, most of the older male adults (more than 95%) reported that they had a single change in occupation during the study period. When we performed a secondary analysis after removing the person with multiple changes, the results were similar (social engagement fully adjusted model; 1.19 (95% CI, 1.02–1.40) for ‘consistently employed’ and 1.34 (95% CI, 1.06–1.70) for ‘unemployed-employed’). Third, there is a lack of information about whether the unemployed participants were in fact seeking occupational opportunities in this study. According to the Ministry of Health and welfare (2008), however, more than 30% of unemployed older adults want and need to work mainly because of economic difficulties. Within this context, the need of employment for older adults has been emphasized. Fourth, this study sample was drawn from a representative sample of Koreans, but the population we focused on was older men. These findings might not be applicable to other subpopulations due to large differences in their life course. In Korea, older men generally pursued an occupation, shouldered the responsibility for the family economy, and reached retirement at a certain age. The life course of these men may provide a positive attitude for working when they reach old age, and they may be familiar with working. In contrast, most women have devoted themselves to family life and have largely assumed their expected role as a homemaker. As women reach old age, they may show a relatively passive attitude toward working. This difference between the sexes may result in different determinants of occupational changes; thus, the current study included only men in its study population. Another limitation is that evaluations on social engagement, depressive symptoms, and the presence of chronic diseases were done by brief questionnaires. We did not include the details (i.e., frequencies, types) of social engagement or clinical information on depression or chronic diseases in this analysis. The addition of these variables could affect our observed findings. Additionally, the possibility of omitted variable bias and heterogeneity within the four categories of occupational status is likely.

Finally, our finding suggests that social engagement and health status are likely to affect opportunities to continue working or to start working for older male adults. We believe that identifying individual components affecting employment and/or unemployment among older adults will be essential for preparing for their golden years and establishing a policy direction for the elderly.

## References

[1] Jackson J, Carlson M, Mandel D, Zemke R, Clark F (1998) Occupation in lifestyle redesign: the Well Elderly Study Occupational Therapy Program. American Journal of Occupational Therapy 52: 326–336. 9588257 10.5014/ajot.52.5.326

[2] Hansson RO, DeKoekkoek PD, Neece WM, Patterson DW (1997) Successful aging at work: Annual review, 1992–1996: The older worker and transitions to retirement. Journal of Vocational Behavior 51: 202–233.

[3] Buck H, Dworschak B (2003) Ageing and work in Europe. Strategies at company level and public policies in selected European countries. Stuttgart.

[4] Frese M, Mohr G (1987) Prolonged unemployment and depression in older workers: a longitudinal study of intervening variables. Soc Sci Med 25: 173–178. 3660007 10.1016/0277-9536(87)90385-6

[5] Dartigues JF, Gagnon M, Letenneur L, Barberger-Gateau P, Commenges D, et al (1992) Principal lifetime occupation and cognitive impairment in a French elderly cohort (Paquid). Am J Epidemiol 135: 981–988. 1595696 10.1093/oxfordjournals.aje.a116410

[6] Andel R, Kåreholt I, Parker MG, Thorslund M, Gatz M (2007) Complexity of primary lifetime occupation and cognition in advanced old age. J Aging Health 19: 397–415. 17496241 10.1177/0898264307300171

[7] Grundy E, Glaser K (2000) Socio-demographic differences in the onset and progression of disability in early old age: a longitudinal study. Age Ageing 29: 149–157. 10791450 10.1093/ageing/29.2.149

[8] Russo A, Onder G, Cesari M, Zamboni V, Barillaro C, et al (2006) Lifetime occupation and physical function: a prospective cohort study on persons aged 80 years and older living in a community. Occup Environ Med 63: 438–442. 16782827 10.1136/oem.2005.023549 PMC2092516

[9] McGarry K (2004) “Health and retirement: do changes in health affect retirement expectations?”. Journal of Human Resources 39: 624–648.

[10] Mein G, Martikainen P, Hemingway H, Stansfeld S, Marmot M (2003) Is retirement good or bad for mental and physical health functioning? Whitehall II longitudinal study of civil servants. J Epidemiol Community Health 57: 46–49. 12490648 10.1136/jech.57.1.46 PMC1732267

[11] Gallo WT, Bradley EH, Siegel M, Kasl SV (2000) Health effects of involuntary job loss among older workers: findings from the health and retirement survey. J Gerontol B Psychol Sci Soc Sci 55: S131–140. 11833981 10.1093/geronb/55.3.s131

[12] Bath PA, Deeg D (2005) Social engagement and health outcomes among older people: introduction to a special section. Eur J Ageing 2: 24–30. 28794713 10.1007/s10433-005-0019-4 PMC5547666

[13] Berkman LF, Glass T, Brissette I, Seeman TE (2000) From social integration to health: Durkheim in the new millennium. Soc Sci Med 51: 843–857. 10972429 10.1016/s0277-9536(00)00065-4

[14] Berkman LF (1995) The role of social relations in health promotion. Psychosom Med 57: 245–254. 7652125 10.1097/00006842-199505000-00006

[15] KLoSA website. Available: http://www.kli.re.kr/klosa/ko/main/main.jsp. Accessed 2012 Sept 9.

[16] Irwin M, Artin KH, Oxman MN (1999) Screening for depression in the older adult: criterion validity of the 10-item Center for Epidemiological Studies Depression Scale (CES-D). Arch Intern Med 159: 1701–1704. 10448771 10.1001/archinte.159.15.1701

[17] Bassuk SS, Glass TA, Berkman LF (1999) Social disengagement and incident cognitive decline in community-dwelling elderly persons. Ann Intern Med 131: 165–173. 10428732 10.7326/0003-4819-131-3-199908030-00002

[18] Barnes LL, Mendes de Leon CF, Wilson RS, Bienias JL, Evans DA (2004) Social resources and cognitive decline in a population of older African Americans and whites. Neurology 63: 2322–2326. 15623694 10.1212/01.wnl.0000147473.04043.b3

[19] Krueger KR, Wilson RS, Kamenetsky JM, Barnes LL, Bienias JL, et al (2009) Social engagement and cognitive function in old age. Exp Aging Res 35: 45–60. 19173101 10.1080/03610730802545028 PMC2758920

[20] Raji MA, Kuo YF, Snih SA, Markides KS, Peek MK, et al (2005) Cognitive status, muscle strength, and subsequent disability in older Mexican Americans. J Am Geriatr Soc 53: 1462–1468. 16137273 10.1111/j.1532-5415.2005.53457.x

[21] Mendes de Leon CF, Glass TA, Beckett LA, Seeman TE, Evans DA, et al (1999) Social networks and disability transitions across eight intervals of yearly data in the New Haven EPESE. J Gerontol B Psychol Sci Soc Sci 54: S162–172. 10363047 10.1093/geronb/54b.3.s162

[22] Bennett KM (2002) Low level social engagement as a precursor of mortality among people in later life. Age Ageing 31: 165–168. 12006303 10.1093/ageing/31.3.165

[23] Mendes de Leon CF, Glass TA, Berkman LF (2003) Social engagement and disability in a community population of older adults: the New Haven EPESE. Am J Epidemiol 157: 633–642. 12672683 10.1093/aje/kwg028

[24] Glass TA, De Leon CF, Bassuk SS, Berkman LF (2006) Social engagement and depressive symptoms in late life: longitudinal findings. J Aging Health 18: 604–628. 16835392 10.1177/0898264306291017

[25] Isaac V, Stewart R, Artero S, Ancelin ML, Ritchie K (2009) Social activity and improvement in depressive symptoms in older people: a prospective community cohort study. Am J Geriatr Psychiatry 17: 688–696. 19625786 10.1097/JGP.0b013e3181a88441

[26] Buchman AS, Boyle PA, Wilson RS, Fleischman DA, Leurgans S, et al (2009) Association between late-life social activity and motor decline in older adults. Arch Intern Med 169: 1139–1146. 19546415 10.1001/archinternmed.2009.135 PMC2775502

[27] Cohen S, Gottlieb BH, Underwood LG (2000) Social Relationships and Health. In: Cohen S, Underwood LG, Gottlieb BH, editors. Measuring and intervening in social support. New York: Oxford University Press. 3–25.

[28] Rantanen T, Guralnik JM, Foley D, Masaki K, Leveille S, et al (1999) Midlife hand grip strength as a predictor of old age disability. JAMA 281: 558–560. 10022113 10.1001/jama.281.6.558

[29] Alfaro-Acha A, Al Snih S, Raji MA, Kuo YF, Markides KS, et al (2006) Handgrip strength and cognitive decline in older Mexican Americans. J Gerontol A Biol Sci Med Sci 61: 859–865. 16912105 10.1093/gerona/61.8.859 PMC1635471

[30] Rantanen T, Masaki K, Foley D, Izmirlian G, White L, et al (1998) Grip strength changes over 27 yr in Japanese-American men. J Appl Physiol 85: 2047–2053. 9843525 10.1152/jappl.1998.85.6.2047

[31] Al Snih S, Markides KS, Ray L, Ostir GV, Goodwin JS (2002) Handgrip strength and mortality in older Mexican Americans. J Am Geriatr Soc 50: 1250–1256. 12133020 10.1046/j.1532-5415.2002.50312.x

[32] Taekema DG, Gussekloo J, Maier AB, Westendorp RG, de Craen AJ (2010) Handgrip strength as a predictor of functional, psychological and social health. A prospective population-based study among the oldest old. Age Ageing 39: 331–337. 20219767 10.1093/ageing/afq022

[33] Kiosses DN, Klimstra S, Murphy C, Alexopoulos GS (2001) Executive dysfunction and disability in elderly patients with major depression. Am J Geriatr Psychiatry 9: 269–274. 11481135

[34] Wilson RS, Mendes De Leon CF, Bennett DA, Bienias JL, Evans DA (2004) Depressive symptoms and cognitive decline in a community population of older persons. J Neurol Neurosurg Psychiatry 75: 126–129. 14707321 PMC1757443

[35] Black SA, Markides KS (1999) Depressive symptoms and mortality in older Mexican Americans. Ann Epidemiol 9: 45–52. 9915608 10.1016/s1047-2797(98)00025-8

[36] Reblin M, Uchino BN (2008) Social and emotional support and its implication for health. Curr Opin Psychiatry 21: 201–205. 18332671 10.1097/YCO.0b013e3282f3ad89 PMC2729718

